# A role for cardiopulmonary exercise testing in detecting physiological changes underlying health status in Idiopathic pulmonary fibrosis: a feasibility study

**DOI:** 10.1186/s12890-021-01520-8

**Published:** 2021-05-05

**Authors:** R. Davis, C. Dixon, A. B. Millar, N. A. Maskell, S. L. Barratt

**Affiliations:** 1 Academic Respiratory Unit, School of Clinical Sciences, Southmead Hospital, University of Bristol, Learning and Research Building, Bristol, BS10 5NB UK; 2 Bristol Interstitial Lung Disease Service, North Bristol NHS Trust, Bristol, UK

**Keywords:** Cardiopulmonary exercise testing, Idiopathic pulmonary fibrosis, Health status

## Abstract

**Introduction:**

There is limited data available on the use of CPET as a predictive tool for disease outcomes in the setting of IPF. We investigated the feasibility of undertaking CPET and the relationship between CPET and quality of life measurements in a well-defined population of mild and moderate IPF patients.

**Methods:**

A prospective, single-centre observational study.

**Results:**

Thirty-two IPF patients (mild n = 23, moderate n = 9) participated in the study, n = 13 mild patients attended for repeat CPET testing at 12 months. At baseline, total K-BILD scores and total IPF-PROM scores significantly correlated with 6MWT distance, but not with baseline FVC % predicted, TLco % predicted, baseline or minimum SpO_2_. VO_2_ peak/kg at AT positively correlated with total scores, breathlessness/activity and chest domains of the K-BILD questionnaire (*p* < 0.05). VO_2_ peak significantly correlated with total IPF PROM scores and wellbeing domains (*p* < 0.05), with a trend towards statistical significance for total IPF-PROM and VO_2_ peak/kg at anaerobic threshold (*p* = 0.06).

There was a statistically significant reduction in FVC% predicted at 12 months follow up, although the mean absolute decline was < 10% (*p* < 0.05). During this period VO_2_ peak significantly reduced (21.6 ml/kg/min ± 2.9 vs 19.1 ± 2.8; *p* = 0.017), with corresponding reductions in total K-BILD and breathlessness/activity domains that exceeded the MCID for responsiveness. Lower baseline VO_2_ peak/kg at anaerobic threshold correlated with greater declines in total K-BILD scores (r =  − 0.62, 0.024) at 12 months. Whilst baseline FVC% predicted or TLco % predicted did not predict change in health status,

**Conclusion:**

We have shown that it is feasible to undertake CPET in patients with mild to moderate IPF.

CPET measures of VO_2_ peak correlated with both baseline and change in K-BILD measurements at 1 year, despite relatively stable standard lung function (declines of < 10% in FVC), suggesting its potential sensitivity to detect physiological changes underlying health status.

**Supplementary Information:**

The online version contains supplementary material available at 10.1186/s12890-021-01520-8.

## Introduction

Idiopathic Pulmonary Fibrosis (IPF) is a progressive fibrosing lung disease of increasing prevalence [1], associated with median survival of only 3–5 years from diagnosis [2, 3]. Disease heterogeneity continues to present challenges for clinicians with regards to prognostication and optimal timings for lung transplantation and/or advanced care planning [4, 5]. In the setting of large-scale clinical trials, a decline in forced vital capacity (FVC) has been used as a primary outcome measure [6, 7] and as a surrogate for mortality, although this has not been universally endorsed [8, 9].

Cardiopulmonary exercise testing (CPET) is the considered the gold standard for evaluating maximal/symptom-limited exercise tolerance, encompassing respiratory, cardiovascular and musculoskeletal assessments, in a controlled laboratory environment [10–12].

However there is limited data available on the use of CPET as a predictive tool for disease outcomes in the setting of IPF. A recent systematic review identified only two small-scale prospective studies that investigated the role of CPET in the prognostication in IPF [13, 14] and concluded that there was insufficient evidence to support its use in facilitating ‘real world’ clinical decisions at the current time.

We have investigated the feasibility of undertaking CPET in a population of mild and moderate IPF patients in terms of the attrition of participants, information on safety data, and willingness to engage with the study protocol. Secondary end-points included: the change in CPET parameters over a 1 year period and the correlation between baseline CPET parameters and change in lung function, 6MWT and health status at 1 year.

We hypothesised that CPET would be feasible in population of mild to moderate IPF patients and more sensitive to change in patient’s health status than 6MWT, FVC or TLco.

## Methods

### Study design

This was a prospective, single-centre observational study undertaken at a large secondary care institution in the UK, providing secondary and tertiary care to patients with Interstitial Lung disease (ILD) within the South-West of England. The study was approved by the Health Research Authority and Research Ethics Committees (IRAS 223450).

### Study subjects

Patients with a multidisciplinary team (MDT) diagnosis of IPF, based upon the American Thoracic Society/European Respiratory Society 2018 guidelines [15], were prospectively recruited to the study between June 2018 and May 2019. Written informed consent was obtained from each patient.

Patients were divided into a ‘mild’ or ‘moderate’ category dependent on their baseline Forced Vital Capacity (FVC ≥ 50% −  < 80%: moderate; FVC ≥ 80% mild). Those patients in the ‘mild’ disease group would undertake both a baseline and repeat CPET at 12 months. It was decided by the study committee, due to the uncertainty of the ability of those with reduced lung function to perform a maximal exercise test, that those in the ‘moderate’ disease group would undertake only a baseline CPET test.

### Inclusion and exclusion criteria

Inclusion criteria were an MDT consensus diagnosis of IPF, male or female aged ≥ 40 years, TLCO ≥ 50% predicted and FVC ≥ 50% with written informed consent for study participation.

Key exclusion criteria were: FEV1/FVC ratio < the lower limit of normal, mobility issues preventing the participant to undertake cycle ergometry, history of myocardial infarction (MI) within 6 months or unstable angina within 1 month, uncontrolled arrhythmias causing symptoms or haemodynamic compromise, history of recent syncope (within last 6 months), acute thrombosis within previous 6 months, cognitive impairment/ inability to perform CPET, severe or untreated arterial hypertension (> 200 mmHg systolic at rest, > 120 mmHg diastolic) and patients using oxygen treatment.

### Participant testing

#### Pulmonary function testing

Pulmonary function tests were performed in accordance with ATS/ERS guidelines [16], using the European Community of Coal and Steel (ECCS) reference equations [17]. Forced expiratory volume during first second of expiration (FEV_1_), forced vital capacity (FVC), and transfer factor for carbon monoxide (TLCO) were undertaken at baseline (within 4 weeks of CPET) and at 12 months (± 4 weeks) (nSpire HDpft, nSpire Health GmbH, Germany). The MRC score, age (years), height (meters), and body weight of the patients (kilograms) were also recorded.

#### 6 minute walking test (6MWT)

A 6MWT was performed at baseline (and within 3 months of CPET) according to ATS guidelines [18], using the Enright reference equation [19]. The following data were collected and analysed: distance achieved (metres), oxygen saturation at the initiation of the test, the minimum saturation level, percentage of theoretical distance achieved and at the end of the test.

#### Cardiopulmonary exercise testing (CPET)

CPET was performed using a standardized protocol in accordance with the American Thoracic Society/American College of Chest Physicians (ATS/ACCP) statement [20], using Wasserman [21] and Jones [22] reference equations. All patients underwent a symptom-limited CPET to exhaustion or intolerability with an electromagnetically braked cycle ergometer (Ergoselect 100, ergoline GmbH, Germany) using a ramp protocol over 8–12 min. The protocol included 3 min of rest, 2 min of unloaded cycling (at 60 revolutions per min), followed by a progressively increasing work rate in a ramp fashion, and a recovery period (patient dependent). The work rate increment for each ramped exercise test was selected depending on the patient’s level of daily activity (either 5 or 10 W/min ramp).

Subjects were asked to maintain a rate of 60 revolutions per minute throughout the exercise period. Several markers were used to determine if a maximal effort test was performed; a respiratory exchange ratio (RER; VCO_2_/VO_2_) ≥ 1.1, maximum heart rate (HR max) > 90% of maximum predicted HR (220-age), maximum minute ventilation during exercise > 85% predicted based on MVV at rest (maximum voluntary ventilation) and a plateau in VO_2_ with an increased workload. CPET could be discontinued at the discretion of the supervising attendant if clinically indicated.

Cardiopulmonary data were collected and analysed with nSpire Zan 600 USB system (nSpire Health GmbH, Germany).

The following parameters were recorded:

- Peak oxygen consumption (VO_2_ peak, ml/kg/min),
- Oxygen consumption at anaerobic threshold
- Carbon dioxide production (VCO_2_)
- Peak minute ventilation (VE peak)(marker of ventilatory function during exercise),
- VE/VCO_2_ slope as derived from the above values (reflects changes in ventilatory drive)
- Peripheral capillary oxygen saturation SpO_2_ (marker of hypoxaemia indicating possible ventilatory limitation to exercise)
- Peak power output (W)(marker of musculoskeletal function)
- Heart rate (HR) (marker of cardiac function during exercise),
- Breathing reserve (BR)

Anaerobic threshold was determined noninvasively through the plot of VCO_2_ versus VO_2_ (V-slope method). Predicted minute ventilation was automatically calculated by the software as a function of maximal voluntary ventilation (MVV), where MVV = FEV1 L × 40. The BR was automatically calculated from the software as the difference between the maximum voluntary ventilation at rest and the peak ventilation. The % predicted VO_2_ peak and % theoretical VO_2_ peak at AT were not determined in this study as it was felt that populations deriving existing reference equations and normal standards were not representative of the male predominant and elderly population, characteristic of IPF patients/populations.

### Health status questionnaires and patient-reported outcomes

Patients were asked to complete the King’s Brief ILD questionnaire (K-BILD) [23] and IPF-Patient reported outcome measure (IPF-PROM) [24], in addition to the Leicester Cough visual analogue scale (VAS) for cough [25] and Bristol VAS for breathlessness and fatigue [26], at baseline and at 12 months.

The K-BILD is a self-completed health status questionnaire that comprises 15 items in three domains of psychological, breathlessness and activities and chest symptoms. The K-BILD scoring system implements logit transformation of raw item response scores to provide total score ranges of 0–100, where 100 represents best health status. The minimally important clinical difference (MCID) for the logit version of the K-BILD questionnaire is 5 for total K-BILD, 6 for Psychological, 7 for Breathlessness and activities and 11 for Chest symptoms [27].

The IPF-PROM [24] is a self-completed 12 item health status questionnaire that measures the physical and psychological experience of breathlessness; emotional well-being and fatigue. The questionnaire has been validated in terms of face and content validity. The scores range from 12 to 48, where 48 indicates worst health status.

### Outcomes

We wished to study the feasibility of undertaking CPET in a population of mild and moderate IPF patients: the attrition of participants, information on safety data, and willingness to engage with the study protocol. Secondary end-points included: the change in CPET parameters over a 1 year period and the correlation between baseline CPET parameters and change in lung function, 6MWT and health status at 1 year.

### Statistical analysis

Categorical variables are reported as absolute numbers and percentages. Normality of continuous data was initially verified using D’Agostino and Pearson normality test. Mean and standard deviations (SD) were used to describe parametric data; median and interquartile range (IQR, in brackets) for non-parametric data. Differences among two groups were verified by t-test with Welch’s correction for continuous parametric data or Mann–Whitney U for non-parametric data. Fisher’s exact test was used to compare two categorical variables (with small sample size), whilst χ^2^-test was used to compare multiple categorical variables or larger sample sizes. Paired t-tests were used for comparison of parametric variables from baseline to 12 months or Wilcoxon in the case of non-parametric data. Pearson’s correlation was used to determine correlations between parametric variables. Data were analysed using GraphPad Prism version 8.0. A *p* value of < 0.05 was considered statistically significant.

## Results

### Study population

**Fig. 1:**
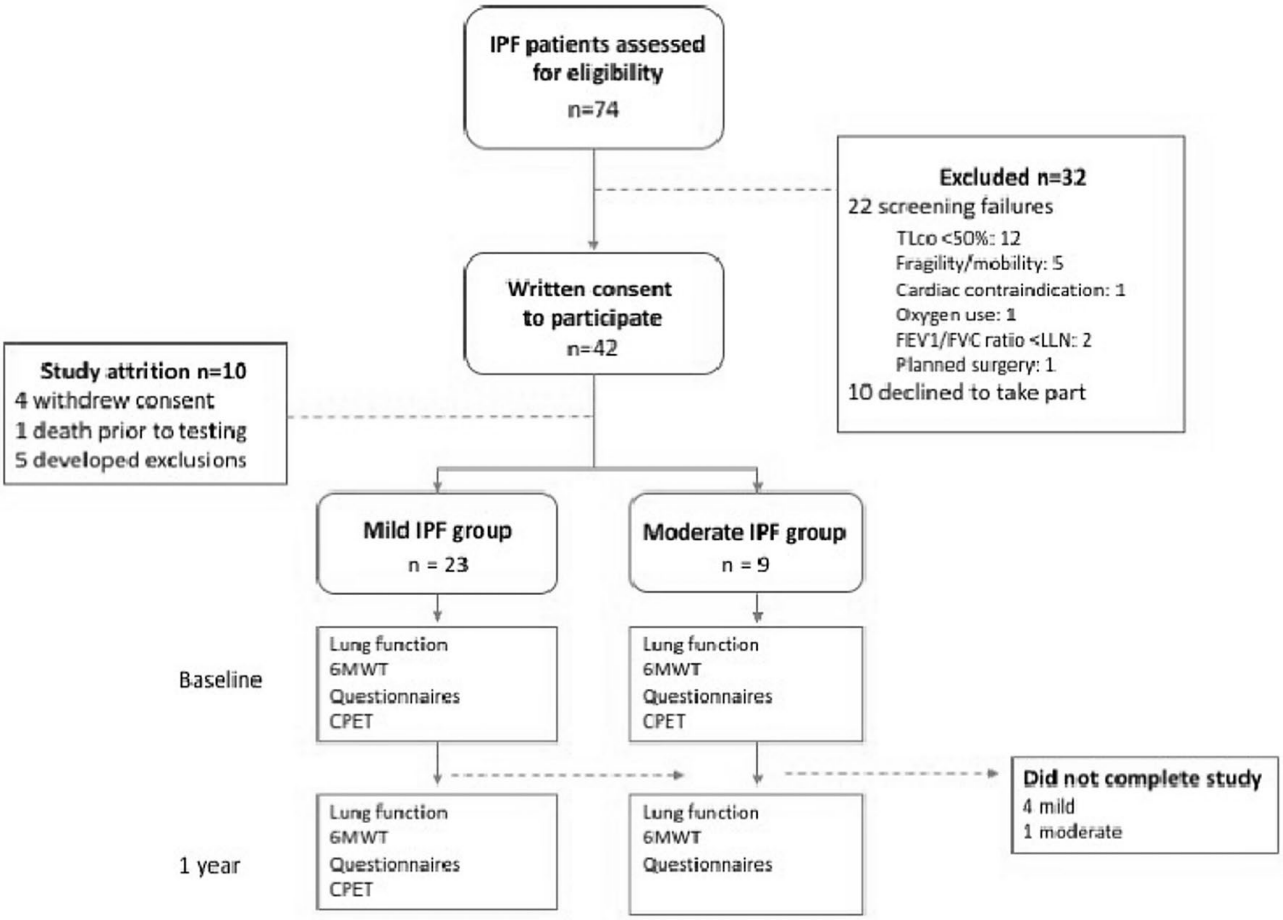
Consort diagram of study participants. TLco_,_ transfer factor; FEV1/FVC < LLN, Forced expiratory volume in 1 s/Forced vital capacity < lower limit of normal; *IPF* idiopathic pulmonary fibrosis; *6MWT* six minute walk test; *CPET* cardiopulmonary exercise testing

Forty-two consecutive IPF patients were prospectively enrolled to the study. Four patients subsequently withdrew consent, 1 patient died and 5 patients developed exclusion criteria prior to commencement of the study. A further 5 patients did not complete the study and were lost to follow up (4 mild, 1 moderate). The final population studied thus consisted of 27 patients (mild n = 19 and moderate n = 8) (Fig. 1). Patients were predominantly male (n = 22, 82%) with a mean age of 75 years (± 6.0 years) and were symptomatic at baseline with a median MRC breathlessness score of 2 (IQR 2–3). Approximately one third (33%, n = 9) of patients (mild n = 5, moderate n = 4) received antifibrotics during the observational period. At completion of 1 year follow up, all patients remained alive.

### Feasibility

There was excellent willingness to engage with the study protocol.

All patients achieved a RER > 1.1 and the vast majority of patients also achieved > 80% of their maximal predicted heart rate (25/27, 93%) and /or had limited breathing reserve, providing corroboration that patients performed at maximal effort.

At baseline, all participants achieved the anaerobic threshold during testing and at 1 year follow up only one patient failed to achieve the anaerobic threshold.

Breathlessness and fatigue were the most commonly cited reasons for terminating CPET. Of patients completing the study (n = 27; 19 mild and 9 moderate IPF), baseline CPET was terminated due to breathlessness in 37% (10/27), the majority of which had mild IPF (90%, 9/10). Leg/muscle fatigue was cited as a reason for terminating CPET in 63% patients (17/27), of which 59% (10/17) had mild disease. There were no significant differences in the reasons for terminating CPET between those that completed and did not complete follow-up.

At 1 year repeat CPET, 54% (7/13) described breathlessness as the reason for stopping and 38% (5/13) muscle fatigue. A dry mouth was cited as the main contributing reason for stopping in one patient.

One patient described dizziness related to his breathlessness during CPET but no other adverse events were recorded. There were no serious adverse events.

### Baseline measurements between mild and moderate IPF groups

Baseline demographics between mild and moderate IPF groups were statistically comparable (Table 1). As per a priori subgroup definitions, participants in the moderate IPF group had a statistically lower baseline FVC % predicted compared to those in the mild IPF group (mild 99% predicted ± 10.0, range 85–125% predicted) vs moderate 70% predicted ± 5.1, range 62–75% predicted, *p* < 0.0001). In keeping with these findings there was a trend towards a lower TLco in those within the moderate IPF group (mild 63% predicted ± 9.5), range 50–83% predicted vs moderate 57% predicted ± 6.2, range 50–65% predicted, *p* = 0.055). No significant difference in the 6MWT distance measured between mild and moderate groups was observed.

**Table 1:** Baseline characteristics of IPF participants

Characteristic	Overall n = 27	Mild n = 19	Moderate n = 8	*p* value
Age (years) (mean, SD)	75 (± 6.0)	75 (± 6.6)	74 (± 4.3)	0.440
Gender (male n, %)	22, 82%	14, 74%	8, 100%	0.280
Smoking history (n, %)				
Current	0, 0%	0, 0%	0, 0%	0.552
Never	10, 37%	9, 47%	1, 12.5%	
Ex-smoker	17, 63%	10, 53%	7, 87.5%	
BMI (kg/m^2^) (mean, SD)	28.5 (± 4.5)	28.9 (± 5.0)	27.7 (± 2.9)	0.585
MRC (n)				0.964
0–1	4	3	1	
2	16	11	5	
3	7	5	2	
4–5	0	0	0	
Antifibrotics (n,%)	9, 33%	5, 26%	4, 50%	0.375
Co-morbidities				n/s
Gastro-oesophageal reflux	11	6	5	
Hypertension	10	7	3	
Coronary artery disease	10	4	6	
Diabetes	3	1	2	
Lung function parameters (mean, SD)				
FVC (L)	2.96 (± 0.72)	3.11 (± 0.78)	2.61 (± 0.46)	0.050
FVC (% predicted)	91 (± 16.0)	99 (± 10.0)	70 (± 5.1)	< 0.0001
FEV1/FVC ratio	78 (± 7.9)	76 (± 7.8)	82 (± 7.2)	0.080
TLco % predicted	61 (± 9.0)	63 (± 9.5)	57 (± 6.2)	0.055
6MWT (mean, SD)	n = 25	n = 18	n = 7	
Distance achieved (m)	350 (± 63.3)	349 (± 66.6)	354 (± 58.6)	0.827
% theoretical distance (m)	78 (± 16.3)	78 (± 16.5)	76 (± 17.0)	0.821
CPET (mean, SD)	n = 27	n = 19	n = 8	
VO_2_ peak/kg (ml/kg/min)*	20.9 (± 3.9)	20.6 (± 4.2)	21.7 (± 3.1)	0.489
VO_2_/kg at AT (ml/kg/min)	13.6 (± 3.5)	13.8 (± 3.6)	13.0 (± 3.2)	0.585
VE peak (L/min)	69.9 (± 21.1)	69.0 (± 21.7)	72.1 (± 20.9)	0.731
VE peak (% predicted)	74.5 (± 14.4)	71.0 (± 13.9)	82.9 (± 12.4)	0.045
VE/VCO_2_ at AT	28.2 (± 3.1)	28.7 (± 3.3)	27.2 (± 2.4)	0.201
Minimum O_2_ saturation during CPET(%)	91 (± 4.7)	91 (± 5.4)	92 (± 2.8)	0.723
Peak work rate (W)	104.8 (± 26.4)	103.6 (± 29.4)	107.6 (± 18.7)	0.673
Peak work (% predicted)	44 (± 8.6)	44 (± 9.5)	43 (± 6.1)	0.565
HR (bpm)	141 (± 21.8)	141 (± 21.2)	139 (± 24.5)	0.793
HR (% predicted)	97 (± 15.1)	98 (± 15.0)	95 (± 16.2)	0.711
BR max (L/min); median (IQR)	26.5 (18.4–32.5)	28.8 (18.8–33.3)	20.4 (12–24.9), n = 7	0.083
K-BILD questionnaire (mean, SD)	n = 27	n = 19	n = 8	
Total	65 (± 9.7)	67 (± 10.3)	60 (± 6.6)	0.058
Psychological domain	69 (± 17.8)	72 (± 17.8)	61 (± 15.7)	0.128
Breathlessness and activity domain	56 (± 12.6)	57 (± 13.6)	52 (± 9.4)	0.267
Chest symptoms domain	78 (± 17.7)	81 (± 18.2)	71 (± 14.6)	0.133
IPF-PROM questionnaire (mean, SD)	n = 27	n = 19	n = 8	
Total	20 (± 4.3)	20 (± 4.6)	21 (± 3.5)	0.337
Physical breathlessness	5 (± 1.5)	5 (± 1.4)	5 (± 1.5)	0.264
Psychological breathlessness	5 (± 1.3)	5 (± 1.2)	6 (± 1.5)	0.121
Well-being	5 (± 2.0)	5 (± 2.2)	5 (± 1.3)	0.662
Energy	5 (± 1.5)	5 (± 1.7)	5 (± 0.8)	0.565
VAS Cough (cm) (median, (IQR))	1.7 (0.8–2.8)	1.5 (0.2–2.6)	2.3 (1.4–3.0)	0.135
Bristol VAS breathlessness (cm)	1.9 (0.8–3.3)	1.8 (0.8–3.4)	2.4 (1.1–4.7)	0.630
Bristol VAS fatigue (cm)	3.7 (1.1–5.1)	3.7 (1.1–5.1)	3.8 (1.4–6.3)	0.457

All data shown as mean with standard deviation(SD) unless otherwise stated. All visual analogue scores presented as median with interquartile range (IQR). *Referenced VO_2_ peak/kg for a 75 year old man would be 26.5 ml/kg/min (based on the linear regression equation male =  − 0.42 × age(years) + 58 (Schneider, Lung 2013 [28]). *years* years; *SD* standard deviation; *n* number; *%* percentage; *FVC* forced vital capacity; *L* litres; *FEV1* forced expiratory volume in one second, *TLco* transfer factor; *m* metres, *HR* heart rate; *MRC* medical research council dyspnoea score; *VAS* visual analogue score; *cm* centimetres; *bpm* beats per minute; *W* Work; *O*_*2*_ oxygen; *L* litres; *6MWT* 6 min walk test; *BMI* body mass index; *CPET* cardiopulmonary exercise test; *K-BILD* King’s Brief interstitial lung disease questionnaire; *IPF-PROM* idiopathic pulmonary fibrosis patient reported outcome measure. Unpaired t-test with Welch’s correction was used to compare parametric data of mild and moderate groups, whilst Mann Whitney u was used for non-parametric data. Fisher’s exact test was used to compare categorical data. A *p* < 0.05 was considered statistically significant

Patients with moderate disease had numerically lower total K-BILD, chest symptom and psychological domain scores compared to those with mild disease, although values were not statistically different (total K-BILD mild disease 67 (± 10.3) vs moderate disease 60 (± 6.6), *p* = 0.058). There were no significant differences in VAS scores of cough, breathlessness or fatigue between mild and moderate IPF groups or IPF-PROM measurements.

Whilst baseline CPET values were all within ‘normal’ published ranges [20, 28], peak minute ventilation (% predicted) was significantly higher for those with moderate IPF compared to those with mild disease (mild 71.0% (± 13.9) vs moderate 82.9% (± 12.4, *p* = 0.045) (Table 1).

Of the baseline CPET parameters measured, VO_2_ peak/kg at anaerobic threshold positively correlated with total scores (r = 0.42, *p* = 0.03), breathlessness/activity (r = 0.47, *p* = 0.014) and chest domains (r = 0.44, *p* = 0.02) of the K-BILD questionnaire (Pearson’s correlation). Similarly, total IPF PROM scores and wellbeing domains significantly correlated with VO_2_ peak (r =  − 0.43, *p* = 0.02 and r =  − 0.44, *p* = 0.02), with a trend towards statistical significance for total IPF-PROM and VO_2_ peak/kg at anaerobic threshold (*p* = 0.06). VE/VCO_2_ at anaerobic threshold also correlated with total K-BILD score (r = 0.39; *p* = 0.001) at baseline, although there were no significant correlations with the individual domains of the questionnaire or IPF-PROM.

Total K-BILD scores (r = 0.44, *p* = 0.03) and total IPF-PROM scores (r =  − 0.43, *p* = 0.03) both significantly correlated with 6MWT distance, but not with baseline FVC % predicted (Total K-BILD, *p* = 0.14; Total IPF-PROM *p* = 0.50), TLco % predicted (Total K-BILD *p* = 0.16; Total IPF-PROM *p* = 0.32), baseline or minimum SpO_2_ (Total K-BILD *p* = 0.25 and *p* = 0.32, respectively, Total IPF PROM *p* = 0.53 and *p* = 0.55, respectively). There were no significant correlations between baseline CPET parameters and VAS scores (*p* > 0.05).

### Measurements at 1 year follow up

#### Total IPF cohort

At one year of follow up, the mean reduction in FVC and TLco % predicted for the whole IPF cohort (n = 27) was − 3.6% (± 7.1, *p* = 0.02) and − 3.2% (± 7.5, *p* = 0.04) respectively (Additional File 1: Table 1). Whilst statistically significant, values were below those deemed clinically significant [29, 30]. There was no significant reduction in 6MWT distance achieved (mean reduction 4.8 m ± 34.7, *p* = 0.50; mean reduction in 6MWT distance as % theoretical distance 0.2% (± 7.9, *p* = 0.92). There was a statistically significant reduction in the breathlessness and activity domain scores of the K-BILD questionnaire from baseline to 1 year, suggesting worse health status (− 4.8 ± 10.2, *p* = 0.02), although this did not reach the published MCID for responsiveness for this domain [27]. There were no statistically significant differences in the VAS scores for cough, breathlessness and fatigue or in the IPF-PROM from baseline to one year (*p* > 0.05).

#### Follow up of mild IPF group with repeat CPET

Thirteen patients from the mild IPF group returned for repeat CPET at 1 year; the coronavirus COVID-19 pandemic prohibited the return of the remaining six patients at the one year follow-up time point as planned. All but one patient achieved anaerobic threshold.

Upon repeat CPET testing there were statistically significant declines in the VO_2_ peak (21.6 ml/kg/min ± 2.9 vs 19.1 ± 2.8; *p* = 0.017), VO_2_ peak at AT (14.2 ml/kg/min ± 3.2 vs 11.8 ± 1.6, *p* = 0.044) VE peak (75.3 L/min ± 20.9 vs 66.1 ± 21.6; *p* = 0.007), peak work (106.9 W ± 26.3 vs 90.8 ± 25.9; *p* = 0.022) heart rate response (142.3 bpm ± 24.0 vs 133 ± 22.3; *p* = 0.040) and increased breathing reserve (BRmax) (21.8 L/min (12.4–34.2) vs 33.8 (20.2–55.7); *p* = 0.0002), compared to baseline values (Table 2).

**Table 2:** Baseline and 1 year follow up data for patients within mild group (those with matched tests)

CPET parameters	Baseline (n = 13)	Follow up (n = 13)	*p* value
VO_2_ peak (ml/kg/min)	21.6 ± 2.9	19.1 ± 2.8	0.017
VO_2_ peak at AT (ml/kg/min)	14.2 ± 3.2	11.8 ± 1.6, n = 12	0.044
VE peak (L/min)	75.3 ± 20.9	66.1 ± 21.6	0.007
VE peak % pred	75.5 ± 13.2	65.9 ± 12.2	0.007
VE/VCO_2_ at AT	29.7 ± 3.1	31 ± 4.6, n = 12	0.353
Minimum O_2_ saturation during CPET (%)	91.5 ± 5.5	87.9 ± 6.6, n = 12	0.182
Peak Work (W)	106.9 ± 26.3	90.8 ± 25.9	0.022
Peak Work (% predicted)	44.3 ± 6.9	37.7 ± 8.5	0.002
HR (bpm)	142.3 ± 24.0	133 ± 22.3	0.040
HR (% predicted)	98.7 ± 16.9	91.8 ± 16.8	0.022
BR max (L/min)(median, (IQR))	21.8 (12.4–34.2)	33.8 (20.2–55.7)	0.0002
6MWT			
Distance achieved (m)	346.9 ± 73.8	340.8 ± 72.4	0.563
% theoretical distance (m)	76.4 ± 18.3	76.0 ± 16.8	0.872
Lung function			
FVC % predicted	98.8 ± 8.5	93.4 ± 10.3	0.010
TLco % predicted	62.3 ± 9.4	59.3 ± 11.8	0.161

*VO*_*2*_ *peak* peak oxygen consumption/kg; *AT* anaerobic threshold; *VE* minute ventilation; *VCO*_*2*_ carbon dioxide output; *VCO*_*2*_ ventilatory equivalent for carbon dioxide; *VE/VCO*_*2*_* BR max* breathing reserve; *W* work; *bpm* beats per minute; *%* percentage; *IQR* interquartile range; *n* number; *6MWT* 6 min walk test; *FVC* Forced Vital Capacity; *TLco* transfer factor; *m* metres; *%* percentage; *CPET* cardiopulmonary exercise testing. All values are shown as mean ± standard deviation, unless otherwise stated. Paired t-test was used for parametric data, whilst Wilcoxon matched pairs signed rank test was used for non-parametric data. A *p* < 0.05 was considered statistically significant

There was a statistically significant reduction in FVC% predicted at 12 months, although the mean absolute decline was < 10% (baseline FVC 98.8% predicted ± 8.5 vs follow up FVC 93.4% predicted ± 10.3, *p* = 0.01). In these same patients, statistically significant reductions in breathlessness/activity (− 7.2 ±  − 10.8; *p* = 0.033) and chest (− 9.6 ±  − 15.0; *p* = 0.040) domain scores of the K-BILD questionnaire were observed, with a trend towards statistical significance for reduction in the total B-ILD score (− 5.6 ±  − 10.4; *p* = 0.077) at follow up. Notably, the mean unit change of total K-BILD and breathlessness/activity domain scores exceeded the minimally clinically important difference previously reported (5 and 7 unit change respectively) [27], with 5/13 patients achieving the MCID for total K-BILD score and 8/13 for the breathlessness/activity domains (Table 3).

**Table 3:** Change in K-BILD, IPF-PROM and Visual analogue scores of mild IPF patients with repeat CPET at 1 year follow up

	Unit change (mean, SD) n = 13	*p* value
K-BILD questionnaire		
Total	− 5.6 (± 10.4)	0.077
Psychological domain	− 5.2 (± 18.8)	0.304
Breathlessness and activity domain	− 7.2 (± 10.8)	0.033
Chest symptoms domain	− 9.6 (± 15.0)	0.040
IPF-PROM		
Total	1.5 (± 0.9)	0.109
Physical breathlessness	0.5 (± 1.4)	0.189
Psychological breathlessness	0.8 (± 1.2)	0.044
Well-being	− 0.2 (± 1.2)	0.656
Energy	0.4 (± 1.4)	0.337
VAS Cough (cm) median	− 1.6	0.391
Bristol VAS breathlessness (cm) median	0.0	0.716
Bristol VAS fatigue (cm) median	− 0.1	0.956

Results shown as mean change in questionnaire score with standard deviations (SD), unless otherwise stated. Paired t-test used for parametric data and Wilcoxon matched pairs signed rank test for non-parametric data. *K-BILD* King’s Brief interstitial lung disease questionnaire; *IPF-PROM* Idiopathic pulmonary fibrosis-patient reported outcome measure; *VAS* visual analogue scale; *cm* centimetres. *p* value < 0.05 considered statistically significant

There were no statistically significant differences in the VAS scores for cough, breathlessness or fatigue VAS score from baseline to one year (*p* > 0.05). There was statistically significant worsening in the psychological experience of breathlessness as reported by the IPF-PROM (0.8 ± 1.2, *p* = 0.044), although the clinical significance of this small statistical change is not clear.

Reductions in K-BILD scores observed at 12 months were correlated with baseline CPET measurements. Lower baseline VO_2_ peak/kg at anaerobic threshold correlated with greater declines in total K-BILD scores (r =  − 0.62, 0.024) and psychological domains of the K-BILD at follow up (r =  − 0.63, *p* = 0.022). No other baseline CPET parameters significantly correlated with change in K-BILD score in this small cohort, including peak work rate. Furthermore, there was no significant correlation with the baseline FVC% predicted (*p* = 0.70) or TLco% predicted (*p* = 0.62) and change in K-BILD score (Pearson’s correlation).

## Discussion

CPET is considered the gold standard for evaluating exertional dyspnoea and exercise intolerance in patients with cardiorespiratory conditions [31], yet currently lacks a defined role in the management of ILD [31, 32]. A recent systematic review by our group highlighted the insufficient available evidence to support the use of CPET in disease prognostication in ILD, emphasising that heterogeneity in terms of the ILD populations studied and the retrospective nature of the majority of published studies limited the conclusions that could be drawn [33]. Furthermore, the minimally clinically important differences for CPET parameters in ILD have not been established.

Our study has shown that CPET can be undertaken in both mild and moderate populations of IPF patients, without any significant adverse events, although study attrition was high and complicated by COVID-19 restrictions, such that only 64% patients completing the protocol.

Our prospective data suggests that baseline CPET VO_2_ peak is associated with clinically-meaningful patient-perceived reduction in health status at 1 year, in spite of comparatively stable lung function parameters (< 10% decline in FVC and < 15% decline in TLco). VO_2_ peak is an integrated measure of respiratory, cardiovascular and neuromuscular function [20]. In a progressive disease such as IPF, the finding of reduced exercise performance at one year was not a surprising one. However, results suggest that this reduction was not as a consequence of ventilatory limitation. There was no change in the CPET ventilatory mode and the development of cardiac + / − pulmonary vascular dysfunction was not apparent. One possible explanation might be that patients became more deconditioned with reduced activity levels in response to their perceived worsening of breathlessness. El Nagger et al. [34] have previously shown that VO_2_ peak correlated with health status of IPF patients at baseline as determined by the St Georges questionnaire but longitudinal changes in CPET parameters and associated health status were not explored. In our cohort, VO_2_ peak during exercise correlated with patient reported outcome measures at baseline, it significantly declined at 12 months and also correlated with the change in patient reported health status at 12 months.

Existing literature conflicts as to whether VO_2_ peak might predict disease outcomes in IPF; peak VO_2_ thresholds ranging from < 8.3 to < 14.2 ml/kg/min [13, 14, 35] have been reported to predict mortality in IPF, whilst others studies have failed to identify any significant association [36–38]. Ongoing follow up of our prospective cohort will be used to further study the use of baseline CPET parameters to predict longer-term outcomes in these patients.

It is recognised that this study has limitations. Firstly, and perhaps most importantly, the study was conducted on a relatively small and homogenous sample of patients. This limits the overall generalisability of results, particularly in terms of feasibility of CPET across IPF phenotypes; for example those with exercise induced pulmonary hypertension versus those with relatively normal pulmonary vascular response to exercise, and the risk of Type II error may be relatively high.

The vast majority of patients had mild IPF (72%) with a median MRC score of 2; again limiting the generalisability of results. Whilst it would have been preferable to have a broader range of symptomatic patients, exercise in patients with high MRC scores would be very restricted leading to early completion of tests before the limit of pulmonary and cardiovascular systems had been reached [39] and thus negatively influencing the results. A further limitation of the study was that almost a quarter of patients enrolled in the study developed exclusions to CPET or were lost to follow up; a factor that will be helpful to inform power calculations for future studies involving CPET as an outcome measure. It was decided by the study committee in the planning of the protocol that due to the uncertainty of the ability and the safety of those with reduced lung function to perform a maximal exercise test, those in the ‘moderate’ disease group would undertake only a baseline CPET test. In retrospect, it would have been more valuable to undertake repeat CPET on all enrolled participants. With the experience gained from this study, this is something that could be explored in the future.

It is recognised by the authors that the selection of reference value equations can have a significant impact on the interpretation of CPET results [20], for example, the 220-age equation may underestimate HRmax in older adults [20]. The authors have used reference equations to contextualise the results where appropriate, recognising that many existing reference equations are not fully representative of the male dominant and elderly population characteristic of IPF populations.

Finally, the COVID-19 pandemic adversely affected the ability to perform follow-up CPET testing, particularly in this highly vulnerable group of individuals; consequently the resulting sample size was small.

## Conclusion

In conclusion, our study provides the initial data to support the feasibility of CPET in at least mild-moderate populations of IPF and the ability of repeated CPET to assess the change in health status over time. This may be clinically applied in the future to assess the response to pharmacological or non-pharmacological interventions from the patient’s perspective. Future work should concentrate on examining the relationship between CPET parameters, lung function and CT-derived measures of disease, establishing the MCID for longitudinal change in CPET in ILD.

## Supplementary Information

**Additional file 1: Table 1**. Change in Lung function, K-BILD, IPF-PROM and Visual analogue scores of all IPF patients at 1 year follow up. Paired t-test or Wilcoxon paired signed rank test.

## Data Availability

The datasets used and/or analysed during the current study available from the corresponding author on reasonable request.
