# Supplementary material for: A role for cardiopulmonary exercise testing in detecting physiological changes underlying health status in Idiopathic pulmonary fibrosis: a feasibility study
Source: BMC Pulm Med. 2021 May 5;21:147. doi: 10.1186/s12890-021-01520-8 (PMC8097115; doi:10.1186/s12890-021-01520-8)
Supplement: Supplementary file 1 — Additional file 1: Table 1. Change in Lung function, K-BILD, IPF-PROM and Visual analogue scores of all IPF patients at 1 year follow up. Paired t-test or Wilcoxon paired signed rank test. [file 12890_2021_1520_MOESM1_ESM.docx]

| **Parameter** | **Change at one year follow up (n=27)** | **P value** |
| --- | --- | --- |
| **Lung function /walk test** | **Mean % change (**± **SD)** |  |
| FVC % predicted | -3.6 (±7.1) | **0.015** |
| TLCO % predicted | -3.2 (±7.5) | **0.037** |
| 6MWT distance % theoretical distance | -0.2 (±7.9) | 0.920 |
| **K-BILD questionnaire** | **Mean unit change (**± **SD)** |  |
| *Total*  *Psychological domain*  *Breathlessness and activity domain*  *Chest symptoms domain* | -2.3 (±1.73)  -1.7 (±16.3)  -4.8 (±10.2)  -2.2 (±14.4) | 0.194  0.590  **0.021**  0.447 |
| **IPF-PROM** |  |  |
| *Total*  *Physical breathlessness*  *Psychological breathlessness*  *Well-being*  *Energy* | 0.5 (±3.2)  0.1 (±1.3)  0.3 (±1.4)  -0.3 (±1.2)  0.4 (±1.1) | 0.408  0.663  0.232  0.164  0.086 |
| VAS Cough (cm) median | -0.2 | 0.601 |
| Bristol VAS breathlessness (cm) median | 0.0 | 0.876 |
| Bristol VAS fatigue (cm) median | -0.1 | 0.925 |

**Supplementary Table 1:** **Change in Lung function, K-BILD, IPF-PROM and Visual analogue scores of all IPF patients at 1 year follow up.** Paired t-test was used for parametric data, whilst Wilcoxon matched pairs signed rank test was used for non-parametric data. A p<0.05 was considered statistically significant. Abbreviations: K-BILD, King’s Brief interstitial lung disease questionnaire; IPF-PROM, Idiopathic pulmonary fibrosis (IPF)-patient reported outcome measure; VAS, visual analogue scale; cm, centimetres; FVC, forced vital capacity; L, litres; FEV1, forced expiratory volume in one second, TLco, transfer factor; 6MWT, six minute walk test.
